# AT_1_-receptor response to non-saturating Ang-II concentrations is amplified by calcium channel blockers

**DOI:** 10.1186/s12872-017-0562-x

**Published:** 2017-05-17

**Authors:** Kristoffer Bernhem, Kalaiselvan Krishnan, Alexander Bondar, Hjalmar Brismar, Anita Aperia, Lena Scott

**Affiliations:** 1 Science for Life Laboratory, Department of Women’s and Children’s Health, Karolinska Institutet, PO Box 1031, 17121 Solna, Sweden; 2 Science for Life Laboratory, Department of Applied Physics, Royal Institute of Technology, Stockholm, Sweden; 3 Institute of Chemical Biology and Fundamental Medicine, 630090 Novosibirsk, Russia

**Keywords:** Calcium, AT1R, Cell imaging, VGCC, hypertension

## Abstract

**Background:**

Blockers of angiotensin II type 1 receptor (AT_1_R) and the voltage gated calcium channel 1.2 (Ca_V_1.2) are commonly used for treatment of hypertension. Yet there is little information about the effect of physiological concentrations of angiotensin II (AngII) on AT_1_R signaling and whether there is a reciprocal regulation of AT_1_R signaling by Ca_V_1.2.

**Methods:**

To elucidate these questions, we have studied the Ca^2+^ signaling response to physiological and pharmacological AngII doses in HEK293a cells, vascular smooth muscle cells and cardiomyocytes using a Ca^2+^ sensitive dye as the principal sensor. Intra-cellular calcium recordings were performed in presence and absence of Ca_V_1.2 blockers. Semi-quantitative imaging methods were used to assess the plasma membrane expression of AT_1_R and G-protein activation.

**Results:**

Repeated exposure to pharmacological (100 nM) concentrations of AngII caused, as expected, a down-regulation of the Ca^2+^ response. In contrast, repeated exposure to physiological (1 nM) AngII concentration resulted in an enhancement of the Ca^2+^ response. The up-regulation of the Ca^2+^ response to repeated 1 nM AngII doses and the down-regulation of the Ca^2+^ response to repeated 100 nM Angll doses were not accompanied by a parallel change of the AT_1_R plasma membrane expression. The Ca^2+^ response to 1 nM of AngII was amplified in the presence of therapeutic concentrations of the Ca_V_1.2 blockers, nifedipine and verapamil, in vascular smooth muscle cells, cardiomyocytes and HEK293a cells. Amplification of the AT_1_R response was also observed following inhibition of the calcium permeable transient receptor potential cation channels, suggesting that the activity of AT_1_R is sensitive to calcium influx.

**Conclusions:**

Our findings have implications for the understanding of hyperactivity of the angiotensin system and for use of Ca^2+^ channel blockers as mono-therapy in hypertension.

## Background

Angiotensin II (AngII) is an important modulator of the sympathetic nervous system, cardiac function, blood pressure and salt excretion. The main receptor of AngII in the cardiovascular system is the angiotensin II type 1 receptor (AT_1_R), which is a G_αq_-protein coupled receptor (GqPCR). Binding of AngII to AT_1_R results in activation of phospholipase C, release of inositol 1,4,5-triphosphate (IP_3_) and Ca^2+^ mobilization from intracellular stores. It is well documented that AngII activation of AT_1_R can be followed by desensitization [1, 2]. The question whether AT_1_R desensitization has clinical implications remains to be resolved, since the majority of cell signaling studies on AT_1_R have been carried out using concentrations of AngII that are at least three orders of magnitude higher than circulating levels [2–6]. Angiotensin receptor blockers and angiotensin converting enzyme (ACE) inhibitors are, along with voltage gated calcium channel (VGCC) blockers, among the most commonly used antihypertensive drugs. These drugs are used alone or in combination [7]. Several lines of evidence suggest that activation of AT_1_R can increase the activity of VGCC [8–10]. Little is however known about the effect of VGCC blockers on the activity of AT_1_R. This is a highly relevant question, since VGCC blockers are sometimes given as mono-therapy.

In the current study, we have compared the AT_1_R signaling pattern in response to repeated application of physiological and pharmacological concentrations of AngII, using a Ca^2+^ sensitive dye as the principal sensor. The effects of physiological concentrations of AngII on the AT_1_R signal were then examined in the presence of the VGCC inhibitors nifedipine and verapamil in therapeutically relevant concentrations. Since there is emerging evidence that some G-protein coupled receptors (GPCR) may be calcium sensitive [11–13], we also determined the effect of physiological concentrations of AngII on the AT_1_R signal in the presence of inhibitors of transient receptor potential cation channels (TRPC), another pathway for Ca^2+^ entry into the cell. The majority of experiments have been performed using a human embryonic kidney cell line 293a (HEK). In order to validate the physiological significance of our findings, key protocols were also performed using rat cardiomyocytes in primary culture.

## Methods

### Cells

Primary rat ventricular cardiomyocytes (RVCM) were obtained from 3 to 5 day old Sprague Dawley (Scanbur, Sollentuna, Sweden) and cultured on 18 mm diameter coverslips for 5 days previously described [14] using a modified growth medium. Growth medium was either a 2:1 mixture of DMEM/F-12:PC-1, supplemented with 2.5% FBS and 0.05 pM of AngII or DMEM for primary cell isolation (Gibco), 1:1000 Cardiomyocyte Growth Supplement (Pierce), 10% FBS and 50 pM AngII. Rats were euthanized by rapid decapitation and the heart removed for generation of cardiomyocyte cultures. Quality of culture was determined using a cardiomyocyte characterization kit (Chemicon). Cardiomyocytes were cultured for 5 days prior to experiment and contracting clusters, seen with transmission light, were selected for recording. Expression of cardiomyocyte markers were confirmed using Troponin I (Chemicon) and Desmin (Chemicon) antibody staining according to manufacturer’s protocol. Rat aortic smooth muscle cells (ASMC, catalog number R6110, 3H Biomedical, ScienCell) were cultured according to manufacturer’s instruction. Briefly, cells were thawed and plated on poly-L-lysine coated coverslips in complete smooth muscle cell medium (SMCM, ScienCell) including 2% FBS and supplemented with 0.05 pM of AngII. Cells were cultured for three to 5 days before experiments, cells from initial plating and first passage were used. Cells were confirmed to express anti-α-smooth muscle actin by immunostaining (antibody from Abcam). Human Embryonic Kidney 293a (HEK, catalog number R70507, Thermo Fisher Scientific) cells were plated on 18 mm diameter coverslips 2 days prior to transfection with Exgen500 (Fermentas), experiments were performed 24 h after transfection. Manufacturers protocols were used for transfection using 1 μg DNA per transfection. HEK cells between passage 3 and 20 was used in experiments. RT-PCR was used to confirm RNA expression of Ca_V_1.2 (Fig 2a).

All experiments were performed according to Karolinska Institutet regulations concerning care and use of laboratory animals, and were approved by the Stockholm North ethical evaluation board for animal research.

### Reagents and constructs

All chemicals and drugs were obtained from Sigma-Aldrich, if not otherwise stated. Bradykinin was purchased from Abcam. 3,5-bis(trifluoromethyl)pyrazole (BTP2) a TRPC channel blocker was obtained from Santa Cruz. Rat angiotensin II type 1 receptor, AT_1_R, with Venus fused at C-terminus was used for Ca^2+^ experiments [15]. The HA-tagged AT1R was made by insertion of PvuI restriction site through mutagenesis in the first extracellular loop between Pro331 and Phe332. Agilent QuikChangeII Site-Directed Mutagenesis kit together with the following primers were used: sense 5′ – GGTGATTGCCGAACGATCGGGGCCAGCGGTAC and antisense 5′ GTACCGCTGGCCCCGATCGTTCGGCAATCACC. The product was digested with PvuI (Thermo Scientific), and RS**YPYDVPDYA**RS (Hemaglutinin flanked by PvuI sites) was inserted through ligation (Phusion Hot Start II, ThermoFisher). Structure of the construct was verified by using BigDye® Terminator V3.1 Cycle Sequencing Kit (Applied Biosystems). pGβ-2A-YFP-Gγ2-IRES-GαqmTq [16] was kindly provided by the lab of Th. W. J. Gadella and used for Gαq-Gβγ FRET (Förster Resonance Energy Transfer) measurements.

### Membrane recruitment

Control and cells exposed to AngII were fixed using 2% paraformaldehyde for 10 min and stained using 1:1000 dilution of Alexa-594 fluorescence conjugated hemagglutinin (HA) antibody (Invitrogen). Cells were imaged using a Zeiss LSM780 in two channels for Venus and Alexa-594 fluorescence. Mean membrane and cytosol fluorescence intensities were calculated. Membrane fraction is reported as normalized to control ratio between mean membrane to cytosolic intensities.

### PCR

Approximately 10^7^ HEK cells were used for RNA extraction using manufacturer’s protocols (RNeasy Plus, Qiagen). cDNA was obtained using iScript cDNA kit (BioRad) following manufacturers protocols. Primers for Ca_V_1.1, Ca_V_1.2 and Ca_V_1.4 were designed and used for PCR amplification (Phusion High-Fidelity DNA polymerase, Thermo Scientific). PCR amplification was run using the manufacturer’s protocols with the following program changes: 40 cycles with 10s extension time. Annealing temperature was 56 °C for Ca_V_1.4, 58 °C for Ca_V_1.1 and Ca_V_1.2. The product was run on a 2% Agarose gel (1:10,000 GelRed, Biotium) and imaged on a Li-Cor Oddyssey system. The following primer pairs were used at 0.5 μM:

CaV11_4689F CAAGAGGAGTATTATGGCTATCGG,

CaV11_5252R GGTCAGCAGTCCCTTCAGCA,

CaV12_6568F CTGCGACATGACCATAGAGG,

CaV12_7188R CAGCGAGGCTCGTACAGA,

CaV14_5045F CAAAGCCACGATGGTCTCCC and.

CaV14_5545R TTTCGCCCACGATGATAGG (Cybergene).

### [Ca^2+^]_i_ measurements

Intracellular Ca^2+^ concentration ([Ca^2+^]_i_) measurements carried out in HEK cells were performed using the fluorescent Ca^2+^ indicator Fura Red (Invitrogen). Cells were loaded with Fura Red using a buffer consisting of 5 μM Fura Red and 1 μl of 20% Pluronic F-127 (Invitrogen) diluted in KREBs (110 mM NaCl, 4 mM KCl, 1 mM NaH_2_PO_4_xH_2_O, 25 mM NaHCO_3_, 1.5 mM CaCl_2_x2H_2_O, 1.25 mM MgCl_2_x6H_2_0, 10 mM Glucose and 20 mM HEPES, pH 7.40, sterile filtered). Cells grown on #1.5 18 mm diameter coverslips (Warner Instruments) were rinsed with KREBs buffer and loaded for 45 min with the loading buffer at 37 °C with 5% CO_2_. After 45 min cells were washed again with KREBs prior to being mounted in a perfusion chamber (Chamlide). Through the use of heated optical table and in-line perfusion feed heater the cells were kept at 37 °C throughout experiments. Loading protocol was evaluated by testing different loading times, both at room temperature and in incubator. The minimal dye concentration usable was identified through Ca^2+^ calibration of serial dilution of dye.

Ca_V_1.2 inhibitors, nifedipine and verapamil, were both mixed fresh for each experimental day. Solutions were kept in fridge and protected from light. We chose to use a high concentration of nifedipine, 100 μM, to compensate for loss of active drugs during the longer experiments. Degradation was unavoidable within each experiment with cells and solutions kept at 37 °C. UV and 488 nm laser excitation also contributed to some drug degradation throughout experiments.

[Ca^2+^] calibration was done by the following protocol, first a 3 min long washout of the KREBs buffer using a 0 [Ca^2+^] containing buffer (110 mM NaCl, 4 mM KCl, 1 mM NaH_2_PO_4_xH_2_O, 25 mM NaHCO_3_, 1.25 mM MgCl_2_x6H_2_0, 250 μM EGTA, 10 mM glucose and 20 mM HEPES, pH 7.40, sterile filtered) followed by 1 μM ionomycin in 0 [Ca^2+^] buffer for 4 min. Calibration ended with 7 min of 1.5 mM [Ca^2+^] KREBs buffer perfusion.

The strongest AT_1_R-expressing cells were excluded from the analysis. Cells not responding to the first stimuli were also excluded. Cells were individually selected and the mean Ca^2+^ dye intensity was calculated and calibrated using the data from the end of each experiment. The mean intensity ratio, R, between the 488 nm excitation image and the 405 nm excitation image for each cell was calculated for each timepoint. R_min_ and R_max_ are the minimum and maximum intensity ratios obtained during calibration. Calibrated [Ca^2+^]_i_, was calculated with k_d_ provided by Invitrogen as:

$$ {\left[{Ca}^{2+}\right]}_i={k}_d\frac{R-{R}_{min}}{R_{max}- R} $$

Ca^2+^ measurements of RVCM were performed using the fluorescent Ca^2+^ indicator Oregon Green BAPTA-1 (Invitrogen) with the same protocol as described for Fura Red. Oregon Green BAPTA1 was chosen as its weaker binding affinity compared to Fura Red made it insensitive to background Ca^2+^ sparklets in RVCM. Calibration was not possible for the RVCM Ca^2+^ experiments, thus the non-calibrated Ca^2+^ response for each cell was obtained by the mean intensity within each selected cell.

Nifedipine effects on bradykinin activation of bradykinin B2 receptors were evaluated through comparing signaling strength with or without pretreatment of 100 μM nifedipine on 100 nM bradykinin (Abcam) treatment. Experiments were performed on Oregon Green Bapta 1 loaded HEK cells. Cells were loaded in the same manner as for Fura Red but with 5 μM Oregon Green BAPTA-1. All other parameters were kept identical as for the 1 nM AngII experiments described above. Each experiment was calibrated as per the protocol described above.

Peak amplitudes were in all experiments calculated as peak above baseline. Baseline was defined as the mean intensity of a stable part of the trace prior to the peak. This calculation reflects the cytosolic increase in concentration rather than the absolute concentration.

### Gαq dissociation

HEK cells expressing AT_1_R and Gαq-Gβγ FRET sensor were exposed to 1 nM AngII with or without 3 min pretreatment of 100 μM nifedipine. FRET was measured using a Zeiss LSM 510 LIVE microscope with a 40×/1.2 water immersion objective and excitation of 405 nm. YFP signal was corrected for mTurquoise bleedthrough and used as FRET intensity. Transfecting HEK cells with GαqmTq allowed for determination of donor bleed through into FRET channel. Bleed through coefficient, *b*, was calculated as I_mTq_/I_FRET_. In experiments, FRET channel intensities were then corrected as: FRET_corrected_ = I_FRET_ – b*I_mTq_. FRET ratios were finally calculated as I_mTurquoise_ / I_FRETcorrected_. Increase in FRET ratio corresponds with increased G_q_-protein dissociation.

### Statistical analysis

Statistical analysis was performed using Matlab, one way Anova analysis and subsequent multiple comparison tests based on studentized range distribution. Quantified data is shown as mean value +/− SEM.

## Results

### AT_1_R desensitization is dose-dependent

**Fig. 1:**
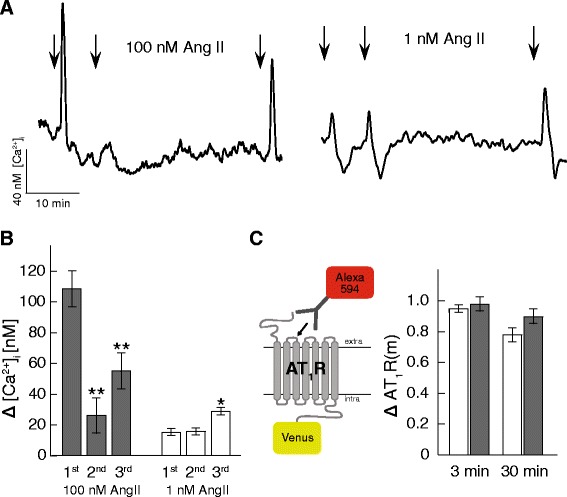
Physiological and pharmacological AngII results in significantly different Ca^2+^ signaling pattern. **a** Representative [Ca^2+^]_i_ traces from Fura Red loaded HEK cells treated with repeated 1 nM (left) or 100 nM (right) AngII for two minutes at times indicated by the arrows, with 5 min recovery after the first exposure and 30 min after second exposure. **b** Quantification of peak [Ca^2+^]_i_ response to repeated AngII treatment 1 nM (open bars) and 100 nM (grey bars) as shown in A. Responses are calculated as peak signal above baseline. *: *p* ≤ 0.05 compared to 1st AngII exposure, **: *p* ≤ 0.01 compared to 1st AngII exposure. *n* = 4 - 8 experiments. **c** The cartoon shows the principle used to detect AT_1_R in plasma membrane. Signal from Anti-HA-Alexa594 antibody show plasma membrane AT_1_R, Venus signal show the total abundance of AT_1_R. Bars show relative change of plasma membrane AT_1_R abundance 3 min and 30 min after exposure to AngII, 1 nM AngII open bars and 100 nM grey bars. Data is normalized to untreated control membrane fraction. *n* = 5 experiments

The majority of studies on AT_1_R signaling have used AngII concentrations that are at least three orders of magnitude higher than the circulatory levels [1, 2, 17, 18], which range between 20 and 100 pM [5]. Repeated exposure to non-physiological AngII concentrations have consistently been shown to result in desensitization of the signaling response. Rat renal interstitial fluid has been reported to reach 3-5 nM AngII under pathological conditions [19–21]. Here we have compared the effect of doses of 1 nM and 100 nM AngII on the calcium signal from HEK cells expressing Venus tagged AT_1_R and loaded with the Ca^2+^ sensitive dye Fura Red. Cells were exposed to three consecutive AngII doses, each 2 min long. The time delay between the 1st and 2nd exposure was 5 min, a common timescale for studies of desensitization [6, 17]. The 3rd exposure to AngII was performed 30 min after the 2nd exposure; a delay selected to test recovery of receptor signaling. Representative [Ca^2+^]_i_ responses to serial AngII exposure are shown in Fig. 1a. The 1st exposure to 100 nM AngII resulted in a seven-fold stronger [Ca^2+^]_i,_ response relative the 1st exposure to 1 nM AngII (Fig 1b). The 2nd exposure to 100 nM AngII shows a significant desensitization. In contrast, no desensitization was observed following the 2nd exposure to 1 nM AngII. A partial recovery of the [Ca^2+^]_i_ response to 100 nM AngII was observed following the 3rd exposure. The [Ca^2+^]_i_ response to the 3rd exposure to 1 nM AngII was significantly stronger than the [Ca^2+^]_i_ response following the 1st exposure (Fig 1b). Application of cyclopiazonic acid, an inhibitor of the sarcoendoplasmic reticulum calcium transport ATPase, completely abolished the [Ca^2+^]_i_ response to AngII, indicating that the increase in [Ca^2+^]_i_ is mainly due to release of Ca^2+^ from intracellular stores (data not shown).

Next we examined to which extent the dose-dependent [Ca^2+^]_i_ response to AngII could be attributed to effects on AT_1_R plasma-membrane expression. For this purpose, we expressed AT_1_R with an extracellular hemagglutinin (HA) tag in addition to a Venus tag at the intracellular C-terminus of HEK cells. Cells were fixed and labeled with an Alexa-594 conjugated anti-HA antibody 3 or 30 min after exposure to AngII. The AT_1_R fraction inserted in the plasma membrane was calculated as the ratio between the fluorescence intensity of Alexa-594 (plasma membrane AT_1_R) and Venus (total AT_1_R) (Fig 1c). The effect of repeated exposure to 1 and 100 nM AngII on the fraction of AT_1_R in the plasma membrane were subtle and did not parallel the changes in Ca^2+^ response.

### AT_1_R signaling is upregulated by nifedipine and verapamil

**Fig. 2:**
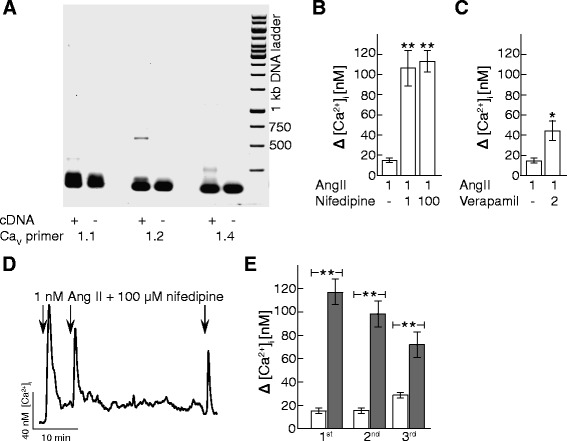
Ca_V_1.2 inhibition up-regulates physiological AT_1_R signaling. **a** Gel image shows a typical RT-PCR of HEK mRNA using primers for Ca_V_1.1(left), Ca_V_1.2 (center) and Ca_V_1.4 (right). As a negative control, each reaction was done omitting the cDNA. Expected band size is, for Ca_V_1.1564 bp, Ca_V_1.2621 bp and Ca_V_1.4501 bp. Gene Ruler 1 kb DNA ladder was used as a size marker. **b** Quantification of peak [Ca^2+^]_i_ response to 1 nM AngII exposure alone or in presence of 1 μM or 100 μM nifedipine in Fura Red loaded HEK cells. *n* = 4 - 6 experiments. **: *p* ≤ 0.01 compared to 1 nM AngII exposure alone. **c** Quantification of peak [Ca^2+^]_i_ response to 1 nM AngII exposure alone or in presence of 2 μM verapamil in Fura Red loaded HEK cells. *n* = 10 experiments. *: *p* ≤ 0.05 compared to 1 nM AngII exposure alone. **d** Representative [Ca^2+^]_i_ traces from Fura Red loaded HEK cells treated with repeated 1 nM AngII for two minutes at times indicated by the arrows in the presence of 100 μM nifedipine. **e** Quantification of peak [Ca^2+^]_i_ response to repeated 1 nM AngII treatment as shown in D. Peaks are calculated as peak signal above baseline. Open bars show 1 nM AngII alone and grey bars show 1 nM AngII in presence of 100 μM nifedipine. *n* = 4 - 6 experiments. **: *p* ≤ 0.01 compared to vehicle

Drugs based on either the nifedipine or verapamil structure, are inhibitors of the L-type VGCC, Ca_V_1.2 subtype [22], and are commonly used in the treatment of hypertension. Since there is emerging evidence of a cross-talk between the Ca_V_1.2 and AT_1_R [8, 23, 24], we tested if nifedipine and verapamil would affect the response to AngII in AT_1_R expressing HEK cells. Ca_V_1.2 is widely expressed, and the RNA expression of Ca_V_1.2 in the HEK cells was confirmed using RT-PCR (Fig 2a). HEK cells were pretreated for 5 min with 1 μM nifedipine or 2 μM verapamil and then exposed to 1 nM AngII for 2 min (Fig. 2b-c). The concentration of nifedipine [25] and verapamil [26] were selected based on human plasma levels. Unexpectedly, we found that in the presence of either drug, there was a robust and highly significant increase of the calcium response to the initial dose of 1 nM AngII in both nifedipine and verapamil treated cells (Fig. 2b and c) To ensure that complete inhibition of Ca_V_1.2 was achieved, as nifedipine is sensitive to heat and light (supplier’s information), we also tested a higher dose, 100 μM nifedipine (Fig 2b). No further increase in response was observed. To eliminate possible errors from drug degradation during the one-hour long protocol we elected to continue with the higher dose of nifedipine.

The effect of nifedipine on the [Ca^2+^]_i_ response to 1 nM AngII was studied using the same three-pulse protocol as described above with the addition of a pretreatment with 100 μM nifedipine (Fig. 2d and e). There was a subsequent reduction of the [Ca^2+^]_i_ response to the 2nd and 3rd dose of 1 nM AngII, suggesting a certain desensitization. There was no increase in the relative distribution of AT_1_R inserted in the plasma membrane in nifedipine pretreated cells compared to control cells (data not shown).

**Fig. 3:**
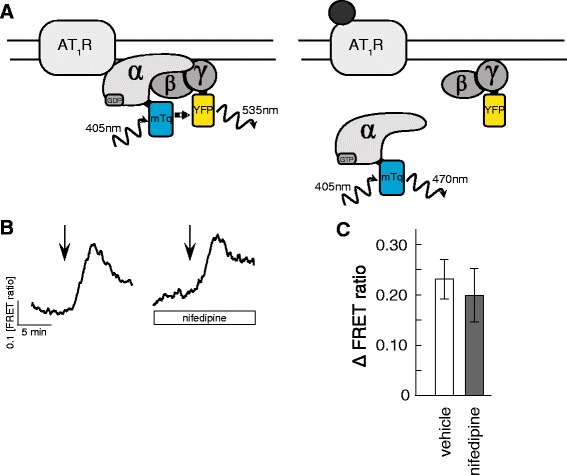
G-protein activation is not affected by nifedipine. **a** The cartoon show the principle of the FRET based assay for detection of G-protein activity. Activity is detected as a decreased FRET signal (increased FRET ratio) when Gαq dissociate from the βγ complex. **b** Representative FRET ratio traces of HEK cells expressing pGβ-2A-YFP-Gγ2 in response to 2 min exposure to (at arrow) 1 nM AngII in absence (left) or presence of 100 μM nifedipine (right). **c** Quantification of peak FRET ratio, in response to 1 nM Ang II alone (open bar) and in presence of nifedipine (grey bar) in HEK cells. *n* = 9 - 11 experiments

We next asked if the nifedipine potentiation of the response to 1 nM AngII could be attributed to increased G-protein activity. To address this question we examined the dissociation of G_αq_ and G_βγ_ subunits induced by AT_1_R activation using a Gαq-Gβγ FRET sensor [16] (Fig 3a). Increased FRET ratio indicates increased G_q_-protein dissociation. The FRET ratio between GαqmTq (donor) and YFP-Gγ2 (acceptor) in response to 1 nM AngII was studied in the presence or absence of nifedipine. No significant difference in FRET ratio was observed between nifedipine treated and control cells (Fig. 3b and c). This suggests that up-regulation of AT_1_R in nifedipine pretreated cells is not due to increased G-protein activation.

### AT_1_R signaling is upregulated by inhibition of TRPC channels

**Fig. 4:**
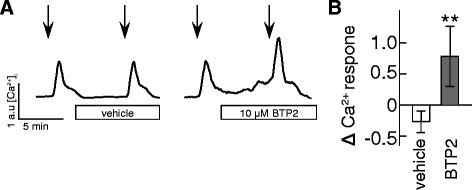
TRPC inhibition up-regulates physiological AT_1_R signaling. **a** Representative [Ca^2+^]_i_ traces from Fura Red loaded HEK cells exposed to 1 nM Ang II (at arrows) followed by 5 min treatment with either vehicle (left) or 10 μM BTP2 (right). **b** Relative change in response between first and second AngII stimulation for vehicle (open bar) and BTP2 (grey bar). *n* = 4 - 5 experiments, **: *p* ≤ 0.01 compared to vehicle

We examined whether the potentiation of the AngII signal observed following Ca_V_1.2 inhibition may also occur following the inhibition of other calcium entry pathways. The TRPC channels are ubiquitously expressed ion channels with high calcium permeability. It was recently reported that the response to AngII was enhanced in arterial myocytes from TRP deficient mice [27]. Here we used BTP2 to inhibit the activity of TRPC channels. BTP2 blocks the calcium influx via TRPC3 and TRPC5 (but not transient receptor potential cation channel subfamily V6) with an IC50 of ~0.1-0.3 μM, and is generally considered to have a many-fold greater potency than other available BTP2 inhibitors [28, 29]. HEK cells were first exposed to 1 nM AngII. This was followed by 5 min treatment with either vehicle or 10 μM BTP2. The cells were then exposed to a second dose of 1 nM AngII (Fig 4a). The relative change in response between first and second AngII exposure was calculated, and showed a significant potentiation of the AngII following BTP2 treatment (Fig 4b).

To test whether the sensitivity for calcium influx observed for the AT_1_R cells is a general phenomenon for GqPCR, we also tested if nifedipine exposure would potentiate [Ca^2+^]_i_ signaling from the bradykinin receptor B2 (B2 receptors), which are constitutively expressed in HEK cells. Exposure of cells to 100 nM bradykinin resulted in similar [Ca^2+^]_i_ response as for 100 nM AngII while in the presence of nifedipine a weaker [Ca^2+^]_i_ response was observed suggesting that not all GqPCRs are potentiated by nifedipine.

### AT_1_R signaling in cardiovascular cells is upregulated by VGCC inhibition

The finding that AT_1_R signaling is enhanced following inhibition of Ca^2+^ influx may have implications for AngII regulation of cardiovascular function, since AT_1_R inhibitors are commonly used to treat cardiovascular disease [30, 31] and Ca_V_1.2 is abundantly expressed throughout arterial and cardiac tissue [32]. We therefore tested if our findings in HEK cells could be reproduced in rat derived primary cultures of both aortic smooth muscle cells (ASMC) and ventricular cardiomyocytes (RVCM).

**Fig. 5:**
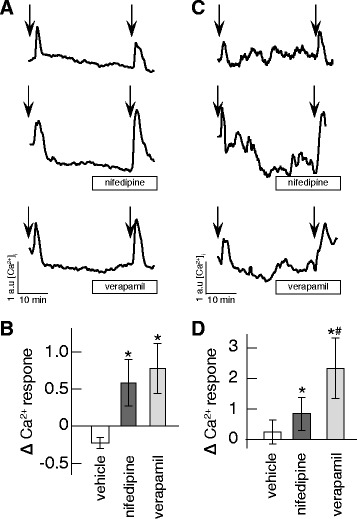
Ca_V_1.2 regulates AT_1_R signaling in rat aortic smooth muscle cells and ventricular cardiomyocytes. (**a**) Representative [Ca^2+^]_i_ traces of Oregon Green Bapta 1 loaded rat aortic smooth muscle cells treated with two minutes of 1 nM AngII at times indicated by arrows. Smooth muscle cells were co-treated from 15 min after first AngII exposure throughout the experiment with vehicle (upper), 0.2 μM nifedipine (middle) and 0.9 μM verapamil (lower). **(b**) Relative change between first and second response to AngII stimulation for vehicle (open bar), 0.2 μM nifedipine (dark grey bar) and 0.9 μM verapamil (light grey bar). Peaks are calculated as signal above baseline. *n* = 6 - 7 individual experiments, *: *p* ≤ 0.05 compared to vehicle. (**c**) Representative [Ca^2+^]_i_ traces of Oregon Green Bapta 1 loaded rat ventricular myocytes treated with two minutes of 1 nM AngII at times indicated by arrows. Myocytes were co-treated from 15 min after first AngII exposure throughout the experiment with vehicle (upper), 0.2 μM nifedipine (middle) and 0.9 μM verapamil (lower). (**d**) Relative change between first and second response to AngII stimulation for vehicle (open bar), 0.2 μM nifedipine (dark grey bar) and 0.9 μM verapamil (light grey bar). Peaks are calculated as signal above baseline. *n* = 6 - 10 individual experiments, *: *p* ≤ 0.05 compared to vehicle, #: *p* ≤ 0.05 compared to nifedipine

ASMC at three to 5 days in vitro expressing alpha-smooth muscle actin were used for these experiments. Cells were loaded with the Ca^2+^ sensitive dye Oregon Green BAPTA1 and exposed to two consecutive physiological doses of AngII thirty minutes apart. Typical [Ca^2+^]_i_ response to 1 nM AngII exposures are shown in Fig. 5a (upper trace). A weaker response was observed at the 2nd exposure, in contrast to what had been found in HEK cells (Fig. 1a-b). We next tested if blocking of Ca_V_1.2 by nifedipine or verapamil would affect the AngII response in ASMC. Nifedipine and verapamil doses were selected based on blood plasma levels reported in patients [33, 34]. ASMC were exposed to 1 nM AngII for 2 min and then allowed 15 min recovery followed by 15 min treatment with vehicle, nifedipine or verapamil and finally exposed to 1 nM AngII for 2 min. Example traces, of the [Ca^2+^]_i_ response in presence of nifedipine and verapamil are shown in Fig. 5a (middle and lower trace). The ratio between the second and first AngII response was calculated. Both nifedipine and verapamil treatment were found to strongly (2-fold) enhance the [Ca^2+^]_i_ response to AngII (Fig 5b) in ASMC.

The same protocol was used to study the effects of VGCC inhibition on AT_1_R signaling in RVCM. RVCM at 5 days in vitro expressing the cardiomyocyte marker Troponin I and displaying contractile activity were used for these experiments. A representative [Ca^2+^]_i_ response trace to 1 nM AngII exposure is shown in Fig. 5c (upper trace). A stronger response was observed at the 2nd exposure, similar to what had been found in HEK cells (see Fig. 1a-b) but in contrast with ASMC response (see Fig 5b). Blocking of Ca_V_1.2 by nifedipine (Fig. 5c middle trace) or verapamil (Fig. 5c bottom trace) were both found to strongly enhance the response to AngII (Fig 5d). Notably, the enhancement by verapamil was significantly stronger than for nifedipine treatment in RVCM but not in ASMC (Fig 5b).

## Discussion

Blockers of the AT_1_ receptor and calcium channel Ca_V_1.2 belong to the most commonly used drugs for treatment of hypertension and cardiovascular disease. Knowledge about AT_1_R signaling is to a large extent based on studies performed with AngII concentrations that exceed physiological and pathophysiological concentrations by several orders of magnitude. There are few studies, if any, that have examined whether Ca_V_1.2 may control the strength of the AT_1_R signal. Here we show that the desensitization of AT_1_R observed following pharmacological concentrations of AngII, does not occur with concentrations of AngII in the physiological range and that acute exposure to therapeutical concentrations of Ca_V_1.2 inhibitors amplify AT_1_R signaling.

AT_1_R is the primary receptor mediating the biological functions of AngII and is abundantly expressed in the cardiovascular and renal systems. AT_1_R signals through release of Ca^2+^ from the intracellular stores via activation of phospholipase C, IP_3_ and the IP_3_ receptor [2]. Both IP_3_ production and Ca^2+^ mobilization are common read-outs of AT_1_R signaling [2, 6, 35, 36]. By performing real-time recordings of [Ca^2+^]_i_, the dynamics of AT_1_R signaling can be monitored continuously in live cell systems, whilst most IP_3_ protocols rely on extraction of IP_3_ at individual time-points. A concern when using Ca^2+^ sensitive dyes is the possibility of toxic effects of the dye and of phototoxicity during prolonged recordings**.** Our protocols were optimized to ensure minimal toxic effects of loading, minimized pixel dwell time and laser power. End point calibrations were performed to ensure that separate measurements are comparable across different systems.

The majority of previous studies on AT_1_R signaling have used AngII concentrations that are several orders of magnitude higher than the circulating and/or tissue levels of AngII [19, 21, 37]. Circulating levels of AngII in healthy humans have been reported to be in the order of 20 pM [4, 5], Patients with heart failure often have several-fold increased levels of AngII [38]. The primary cardiomyocyte cells used in this study were derived from rats. The circulating levels of AngII in rats have been reported to be approximately ten times higher than in humans [19, 37]. Local levels of AngII in tissue can be even higher, depending on local production and metabolism, reaching up to 3-5 nM in rat renal interstitial fluid [19–21].

Homologous desensitization of GPCRs is a well-known phenomenon, and numerous studies of desensitization have been carried out on AT_1_R [1, 2]. Most of these studies have been performed with concentrations of AngII in the range of 10-100 nM. In our study desensitization was only observed after exposure to the high concentration of AngII, while repeated exposure to 1 nM AngII did not result in desensitization. This lack of desensitization to low concentrations of AngII might explain why moderately increased endogenous AngII levels can have a deleterious long-term effect on cardiovascular and renal function.

Voltage-gated calcium channels serve an important role for the control of [Ca^2+^]_i_ homeostasis in almost all mammalian cells and play a particularly dynamic role in excitable cells. The L-type VGCC 2, Ca_V_1.2, is a common target for anti-hypertensive treatment. It was previously reported from several groups that AngII may modify the activity of the Ca_V_1.2 [8–10, 39]. The results are not unambiguous, but the majority of studies have shown that AngII increases the Ca_V_1.2 activity. Here we showed that pre-incubation of both HEK cells and cardiomyocytes with inhibitors of Ca_V_1.2 activity results in a robust amplification of AngII triggered [Ca^2+^]_i_ signal. Notably, this amplifying effect of the Ca_V_1.2 inhibitors was observed with concentrations that are comparable to those used to treat hypertension [25, 26, 33, 34]. The amplification of the AngII signal that we observed in both nifedipine and verapamil treated cells may not be specific for Ca_V_1.2, since it was also found following inhibition of the TRPC channels, which represent another ubiquitous Ca^2+^ influx pathway. We hypothesize that the AT_1_R complex is sensitive to changes in intracellular Ca^2+^ concentration, and that increased Ca^2+^ influx might have a desensitizing effect on the AT_1_R complex. In support of this hypothesis, it has been shown by other groups that signaling from another G-protein coupled receptor, the dopamine D2 receptors, is controlled by intracellular Ca^2+^ concentration [11, 12]. A recent study showed that increased calcium influx through either L-type voltage gated calcium channels or from intracellular stores result in D2 receptor desensitization [13]. The sensitivity of AT_1_R (current study) and D2 receptors [13] to Ca^2+^ influx was not shared by the bradykinin receptor, and is thus not a common phenomenon for all GPCRs. The molecular mechanism by which calcium influx modulates the strength of AT_1_R signaling remains elusive. We found no evidence for a recruitment of receptors to the plasma membrane or altered G_q_-protein activity.

## Conclusions

In conclusion, this study provides novel and clinically relevant information about how inhibition of CaV1.2 upregulate AT_1_R signaling. Notably these effects were observed with physiological and therapeutically relevant concentration of AngII, nifedipine, verapamil. Calcium channel blockers are commonly used to treat hypertension, either in combination with other drugs such as inhibitors of AT_1_R or ACE, or as mono-therapy. When calcium channels blockers are used as mono-therapy, the possibility for stimulation of AT_1_R signaling should be taken into account. Notably, a recent meta-analysis, based on more than 40 studies, has shown that combination therapy that targets both the angiotensin system and the voltage gated calcium channels, will provide a better blood pressure control than mono-therapy that acts at one of these targets [40].

## Abbreviations

ACE: Angiotensin Converting Enzyme
AngII: Angiotensin type II
AT_1_R: Angiotensin II type 1 receptor
BTP2: 3,5-bis(trifluoromethyl)pyrazole
[Ca^2+^]_i_: Intracellular Ca^2+^ concentration
Ca_V_1.2: Voltage gated calcium channel subtype 1.2
DMEM: Dulbecco’s Modified Eagle Medium
FRET: Förster Resonance Energy Transfer
G_αq_: G-protein subunit αq
GPCR: G-protein coupled receptor
GqPCR: G_αq_-protein coupled receptor
G_βγ_: G-protein subunit βγ
HA: Hemagglutinin
HEK: Human Embryonic Kidney cell line 293a
IP_3_: Inositol 1,4,5-triphosphate
RVCM: primary rat ventricular cardiomyocytes
TRPC: transient receptor potential cation channels
VGCC: Voltage Gated Calcium Channel
ASMC: Aortic smooth muscle cells
